# What Goes Around Should Not Move Around: Immobilizing Microplastics as a New Approach for Analytical Ring Trials

**DOI:** 10.1021/acs.est.4c09427

**Published:** 2024-12-03

**Authors:** Robin Lenz, Kristina Enders, Eva Cseperke Vizsolyi, Mareike Schumacher, Julia Lötsch, Martin G. J. Löder, Gabriele Eder, Yuliya Voronko, José Manuel Andrade-Garda, Soledad Muniategui-Lorenzo, Christian Laforsch, Dieter Fischer, Matthias Labrenz

**Affiliations:** 1 Leibniz Institute of Polymer Research Dresden, Dresden 01069, Germany; 2 Leibniz Institute for Baltic Sea Research Warnemünde, Rostock 18119, Germany; 3 University of Bayreuth, Bayreuth 95440, Germany; 4 Österreichisches Forschungsinstitut für Chemie und Technik, Wien 1030, Austria; 5 University of A Coruña, A Coruña 15071, Spain

**Keywords:** spectroscopy, microscopy, particles, colloid, proficiency, validation, round robin, fixation

## Abstract

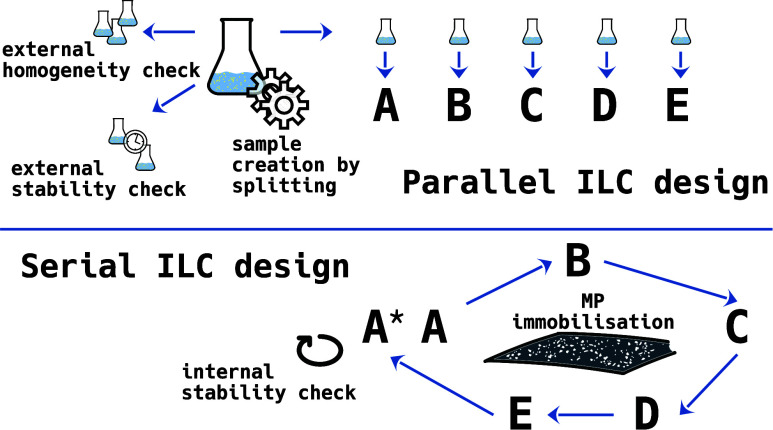

Microplastics have gained importance as pervasive environmental particulate pollutants. Their analysis demands precise quantification methods, with interlaboratory comparisons (ILCs) being crucial for performance assessment. Typically, ILCs follow a parallel design: participants each analyze their own sample specimen, often with significant variability due to challenges in producing identical subsamples of the particulate analyte, inseparably masking the relevant uncertainty sources the ILC intends to measure. We provide a filtration-immobilization approach for particles ≤100 μm, creating permanently immobilized microplastics samples. This enables serial ILC designs where participants sequentially measure the same sample. Demonstrating the concept using 5 polymers immobilized on 10 μm pore-sized silicon filters, we expose the specific measurement uncertainty being 77% lower than the total combined uncertainty observed in a parallel ILC (relative standard deviations: 5 and 23%, respectively). Particle immobilization opens further applications in sample archiving and creation of durable reference samples also for other fields of particulate matter research beyond microplastics.

## Abstract 2

Microplastic quantification standardization is crucial for environmental monitoring, but current interlaboratory comparisons conflate sample preparation and analytical uncertainties. We introduce serial ring trials using immobilized particles to improve analytical uncertainty estimations.

## Introduction

The contamination of the environment with microplastics (MPs), i.e., synthetic polymer particles commonly accepted to be between 1 and 1000 μm,^1^ has created a need for reliable data on the characteristics and quantities of MPs. This has led to a wide variety of methodological developments. The resulting significant differences in resolution and quality of analyses are impeding the comparability of data between studies^2−4^ and more comprehensive environmental risk assessments.^5^

To solve this issue, interlaboratory comparisons (ILCs) play a crucial role in evaluating the reliability and accuracy of methods for the characterization and quantification of MP particles in various samples,^6^ including environmental, food, and tissue matrices. Several ILC studies aimed at MP-analytical proficiency can be found in the recent literature^6−14^ using different sample types such as environmental matrices spiked with specific MP types,^13^ artificial samples such as suspensions,^12^ or MP particles in solid media like tablets or ice cubes.^6,11^ These studies typically follow a parallel design, where each participant receives and analyzes their own sample specimen, assuming equality in MP numbers and composition. This design, based on reporting guidelines like DIN 38402-45:2014-06, as cited by ref (12), ISO 13528,^15^ or the IUPAC technical report,^16^ resembles established procedures for soluble analytes, where homogeneity issues are less of a concern and accurate reference measurements are available. As particulate pollutants, MPs pose significant challenges to this equality assumption, especially for particle sizes in the lower micrometer range where individual handling of particles is impractical.^17^

**Figure 1:**
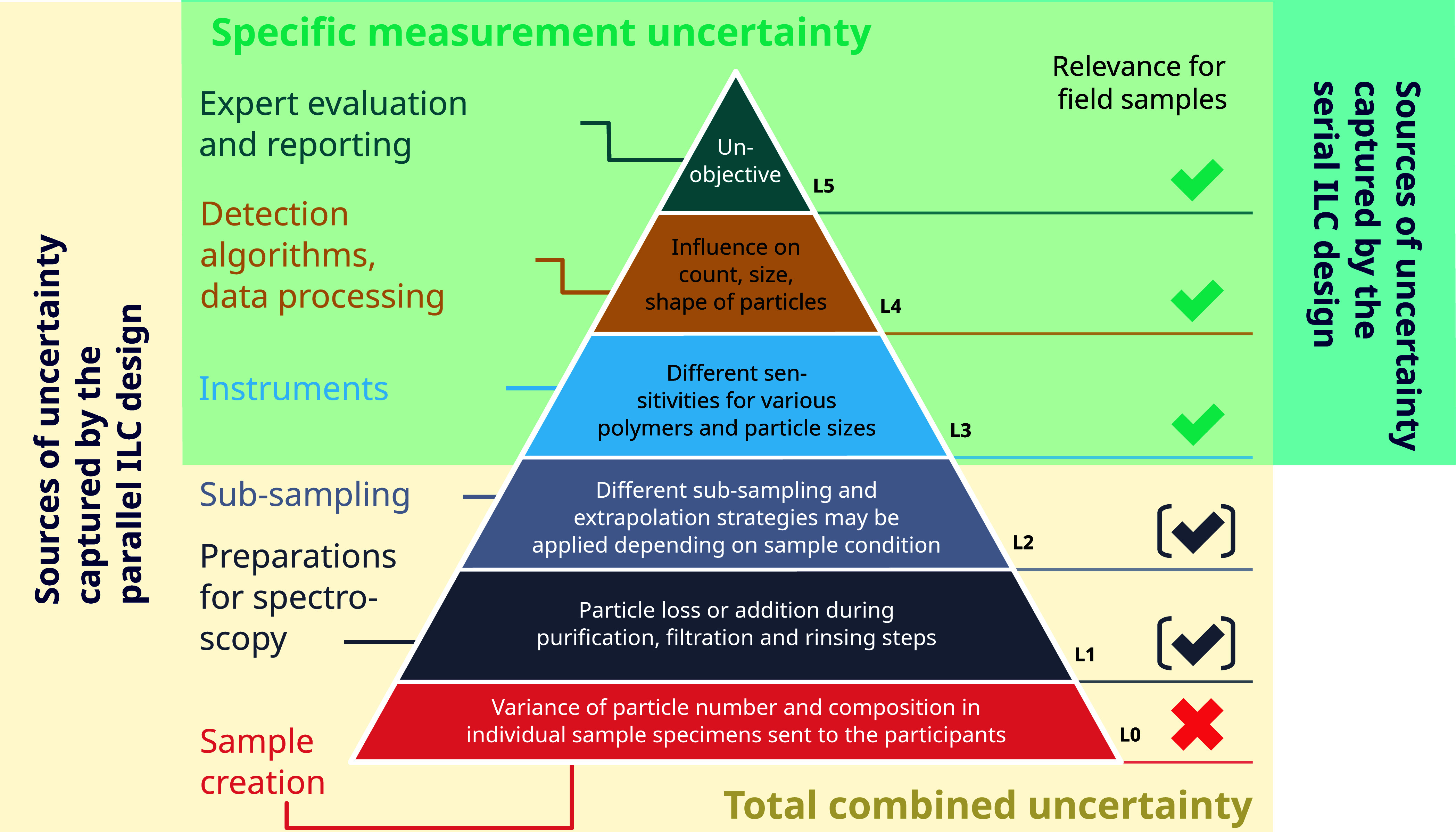
Uncertainties propagating through the pipeline of MP quantification. Colored areas enclose the shares captured by parallel (beige) and serial (mint) ILC design concepts. The relevance of the uncertainty contributors with regard to field samples is shown to the right of the pyramid. It is highlighted that uncertainties arising from the way in which the parallel ILC sample is produced (L0) are irrelevant to real samples and should therefore preferably be excluded by an ILC design. Other uncertainty sources (L1 and L2) can be relevant, but because they depend on the sample conditions, they should be addressed separately from an ILC estimating the specific measurement uncertainty.

The need for comparable and reliable MP quantification led to innovations in creating homogeneous sample specimens for ILCs and reference materials,^11,18−20^ as well as assessment and correction methods^8,21^ and reporting guidelines.^22,23^ Their perpetual integration in new ILCs can improve precision and reduce the uncertainty of the true MP content, gradually making the determination of analytical proficiency more reliable. However, the fundamental problem remains: parallel ILC designs capture compounded effects caused by a range of uncertainty sources whose contributions cannot be further decomposed (Figure 1). At this point, it is important to emphasize the principles of metrology, such as uncertainty propagation according to the “Guide to the expression of uncertainty in measurement” (GUM)^24^ by the Joint Committee for Guides in Metrology (JCGM) whose terminology we apply here to the field of MP analysis.

All steps from planning the sampling to evaluating and reporting the final data contribute to the combined uncertainty in the MP-analytical chain. Therefore, any uncertainties intrinsic to the production of the parallel sample specimens, e.g., from splitting or weighing, are included at the base of the final result (Figure 1). Here, we use the term “specific measurement uncertainty” to describe the combined uncertainty associated with the chemical measurement acquisitions (e.g., microspectroscopy), data evaluation, manual result control, and reporting (Figure 1, L3–L5). In parallel ILCs, the specific measurement uncertainty is inseparable from uncertainty contributions of production and distribution of sample specimens (Figure 1, L0), sample treatments like purification and measurement preparations (Figure 1, L1), and subsampling (Figure 1, L2). The first is specific to the ILC design itself and, as such, not informative about a participant’s proficiency to quantify MP field samples (compare Figure 1, “Relevance for field samples”). The latter two are of potential relevance when considering field samples but may vary depending on the pretreatment requirements of the matrix such as sediments, biota, or food samples. They should, therefore, be addressed by separate QA/QC measures^25^ or a posteriori data alignments.^5^

Currently there is no approach available to investigate the specific measurement uncertainty in ILCs to build the basis for a subsequent evaluation of the total combined uncertainty of the whole sample preparation and analysis process. To address this gap, we adapt the concept of MP particle immobilizations, first described for experiments in the ≤100 μm size fraction,^17,26^ to the requirements of ILCs using an inorganic adhesive. For two common spectroscopic substrate types, porous Si wafers and alumina membranes, we describe an immobilization-by-filtration approach, leading to a permanent fixation of particles on the surface of the carrier. It can be measured sequentially by all ILC participants, with each laboratory measuring the same filter and passing it onto the next participant in the ILC consortium. This methodological paradigm shift renders the ILC literally a “ring trial”, or “round-robin test” as it is also often called, relating to its historic meaning of an institution of coordinated collective activity.^27,28^ We refer to this concept as the serial design to distinguish it from the parallel ILC design. It eliminates the unwanted uncertainty sources introduced by the repetitive sample specimen creation in the parallel design approach. Therefore, the observed variance of the results represents a more accurate magnitude of the specific measurement uncertainty.

## Methods

We conducted a comparison between a parallel design ILC where participants received independent sample specimens of samples P1–P3 and serial ILCs where participants received the same specimens of three different sample types subsequently: S1–S3 (Table 1).

**Table 1:**
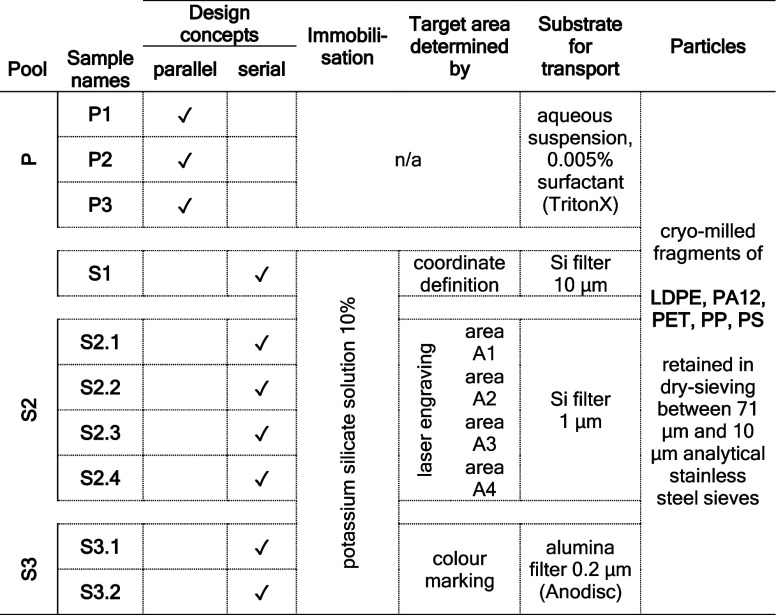
Sample Names and Their Poolings (Pool) that Were Prepared for the Parallel and Serial ILC Designs Are Listed with Their Key Specifications

To encourage broader adoption, we emphasize that the proposed serial ILCs using immobilized particle samples can be conducted using standard laboratory equipment. Any special or custom-made devices, although detailed here for possible replication, can be replaced by commercially available alternatives without compromising the immobilization’s success.

### Sample Preparation

Sample preparation steps were conducted in specialized MP-analytical laboratories under laminar flow benches. As an exception, steps involving handling of dry MP powders such as grinding, mixing, or adding powders to suspension media were conducted outside the laminar flow bench to avoid workplace contamination. White cotton lab coats were used during all procedures.

We obtained the MP particles by sieving five previously cryo-milled (Cryomill, Retsch, Germany) environmentally relevant polymers. The resulting particle shapes were irregular (see also Figure SI5.2). Fibers were not included in this study. We used low-density polyethylene (LDPE), polyamide 12 (PA12), polyethylene terephthalate (PET), polypropylene (PP), and polystyrene (PS). The raw materials and grinding and sieving procedures were identical as reported by a previous study.^17^

The creation of the aqueous suspension samples P1–P3 and the adhesive particle suspension for the immobilized samples S1 and S2 is described in full detail in SI1. In the samples of the serial ILC design (S1–S3), the homogeneity of particle distribution on the filters was not assumed or required, as all participants measured the same defined area of each sample. The selected measurement areas were chosen based on their representativeness of the overall particle immobilization.

#### Immobilization on Si Wafers

Porous silicon wafers were inserted in the precleaned filtration apparatus,^29^ connected to an adjustable vacuum pump (SC920, KNF Neuberger). The device contained red PTFE discs for sealing. For sample S1, a silicon wafer was used (11 mm length and width and 250 μm thickness) with conical pores (15/8 μm terminal diameters and 55 μm interpore distance) supplied by Fraunhofer Institute for Reliability and Microintegration, Moritzburg, Germany; for more details, see ref (30). We refer to it as a nominal 10 μm filter.

For S2, we used a nominal 1 μm pore size filter (Si wafer: 10 mm length and width, 200 μm thickness, cylindrical 1 μm pore diameter, 1.5 μm interpore distance, from SmartMembranes GmbH, Halle, Germany).

The prepared potassium silicate adhesive particle suspension (SI1) was placed on top of a high-frequency excentrical shaker for homogenization, while volumes were being extracted for filtration using 1 mL Pasteur glass pipettes. We applied 2 and 6 mL for S1 and S2, respectively, where particle coverage was found to be suitable on visual inspection. Filtration was conducted at approximately 100 mbar. After curing in a vacuum drying oven (40 °C, 100 mbar, 12 h), filters were flushed with running MP-free water and compressed air to remove any loose particles. This rinsing process was repeated three times. Dark field microscopic images were acquired after each iteration to evaluate the removal of loose material. It was considered complete as no substantial further changes in the particle amounts could be manually observed between the images taken after the second and third rinse.

#### Immobilization on Alumina Filters

The dry powder mixture of MP particles was suspended in MP-free water and filtered onto an Al_2_O_3_ membrane (Anodisc, Whatman, polypropylene outer ring support, 45 μm height, 25 mm diameter, 60 μm membrane thickness, 0.2 μm pore size). The filter was then placed inside a glass Petri dish on top of a droplet of the potassium silicate adhesion medium (SI1), which was allowed to soak through the filter material by capillary rising, wetting the particles from below. Two filters of this type were created and measured, S3.1 and S3.2, with different amounts of MP particles.

#### Target Area Definition

**Figure 2:**
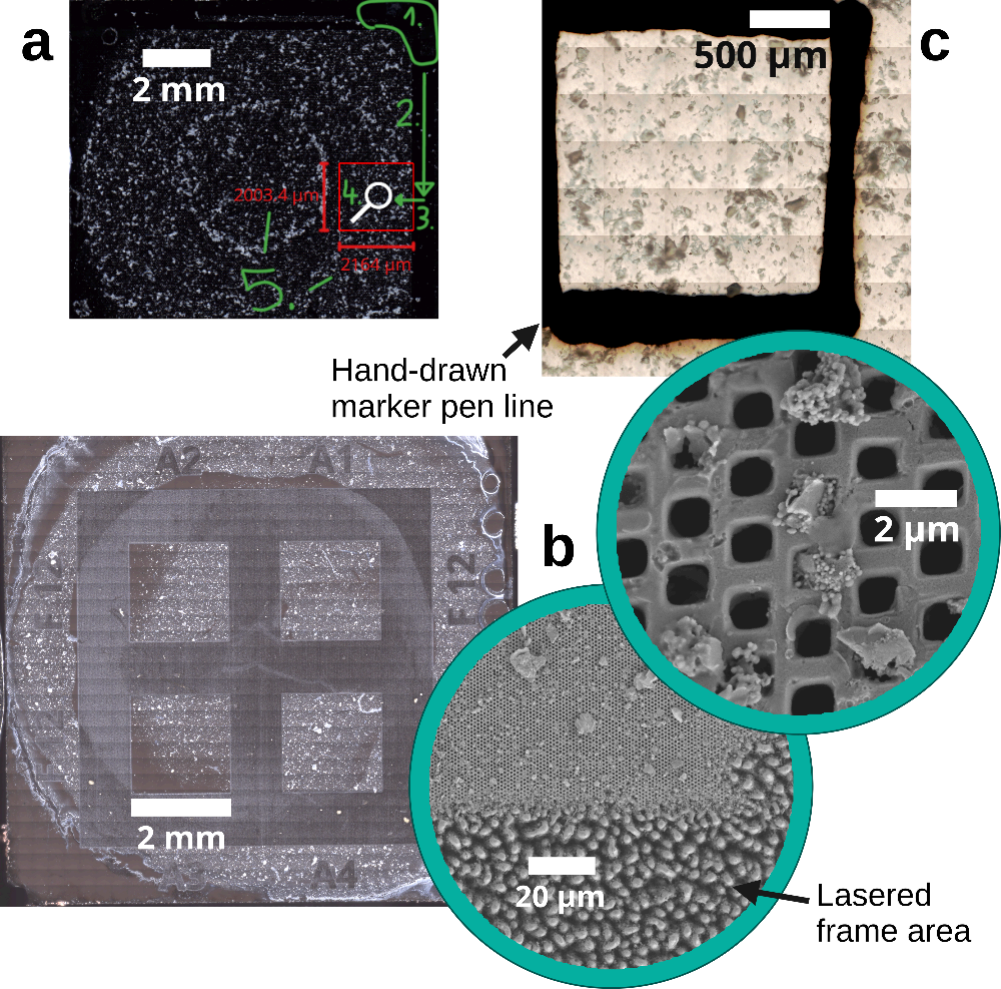
Approaches to defining the measurement area. (a) Dark field image of sample S1 with visual guidelines (green) provided to the participants for manual targeting. (b) Laser-engraved frame and resulting target areas A1–A4 of sample S2 shown as a dark field overview with insets showing SEM images of the area boundaries and the square 1 μm pores. (c) Bright field overview of the Al_2_O_3_ membrane sample S3 with the target area defined by a black marker.

Measuring the entire filter area was not a viable approach for several participants in terms of measurement time and amount of data produced. Instead, rectangular areas of approximately 2 by 2 mm were defined for measurement by each participant. Three approaches to marking the specific target areas were investigated, coordinate definition, laser engraving, and color markings, for samples S1–S3, respectively (see also Table 1 and Figure 2 as well as details and dimensions provided in SI2).

#### Serial ILC Round Trip

The filters were sent to the participants in custom-made filter holders (Figure SI3) by standard mail. In the case of Si filter samples (S1 and S2), participants were advised to rinse the sample with MP-free water and dry it with compressed air prior to measurement to avoid contamination. For the Al_2_O_3_ membrane sample S3, this was not possible due to the fragile nature of the substrate. Al_2_O_3_ membranes provide a less smooth and less even surface than the samples prepared on Si filters. Hence, participants were advised to use adhesive tape on the outer ring support to improve surface flatness during measurement. Participants measured the samples using the method of their choice before repackaging them and sending them onto the next participant. Finally, the samples were received and remeasured by the host participant to assess potential particle losses from the repetitive handling and transport.

### Sample Analysis

The samples from the parallel and serial ILCs were analyzed individually by each participant with no further requirements other than using their laboratory’s state-of-the-art MP analysis techniques and parameters like they would use to analyze field samples. A total of eight participants were involved in the measurements; however, due to the developmental character of this study, not every participant measured every sample and the order of participants varied between samples. Table 2 details the chosen techniques and key parameters of the participants, as well as the samples they measured. A participant in the context of this study is a unique constellation of an institute, an analyst, a measurement instrument, and an analysis software. Participants A–D and G were located all at the same institute and differed only in the technical and analytical choices as described in Table 2.

**Table 2:** ILC Participants with Spectroscopic Techniques and Measured Samples

		**technique**	**instrument**	**measurement parameters**	**software**	**analyzed samples**
participant	A	Raman microscopy	WITec alpha300R	20× objective, 532 nm, 10 mW, 10 × 0.5 s acquisitions	Gepard^31^	all
	B				Witec ParticleScout + Gepard^31^	S1, S2, S3
	C				Gepard^31^ + ML-prototype Raman^a^	S1
						
	D	micro-FTIR imaging	PerkinElmer Spotlight400	transmission, IR range of 4000–650, 8 cm^–1^ resolution, 4 scans/pixel (6.25 μm)	ML-prototype FTIR^a^	all
	E					
	F		Bruker Lumos II	transmission, IR range of 3600–1250, 8 cm^–1^ resolution, 2 scans/pixel (5.7 μm)	Bruker Opus v7.5 + BayreuthMicroplasticsFinder^32^	P1, P2, P3, S2, S3
						
	G	Raman microscopy	Renishaw inVia Qontor	20× objective, 633 nm, 50% power, 7 × 0.5 s acquisitions	Gepard^31^	S1, S2, S3
						
	H	LDIR imaging	Agilent 8700LDIR	quantum-cascade laser IR source, IR range of 1800–975, 8 cm^–1^ resolution, 1 scan/particle	Agilent Clarity software v1.5	S2

a Machine learning-based prototypes for MP quantification developed by Purency GmbH, Vienna, Austria.

#### Common Area Correction

The target area definition of samples S1 and S3 (compare Figure 2a and Figure 2c) resulted in slightly different areas measured by each participant. This was partly due to different rotation angles of the sample on the instrument stages, different image acquisition modes of the instruments, and/or manual inaccuracies. To evaluate results from the same area only, the largest common area was defined by superimposing the obtained particle distribution images in the GNU image manipulation software (https://gimp.org). All images were manually registered to a common coordinate system. A rectangle contained within each image was then defined graphically as the common measurement area. Particles completely outside this common area were counted for each image and polymer type, and their numbers were subsequently subtracted from the original particle counts reported by each participant. The definition of the target area by laser engraving for sample S2 made such a correction unnecessary. The areas were sharply defined and macroscopically visible. As particles in the interstices were burnt off by the laser, a simple measurement of a slightly larger area gave the same particle numbers as measuring an area to its exact extent.

### Statistical Analysis

Results were reported by participants as numbers of particles per polymer type, size range, and sample. Total MPs (TMPs) were calculated as the sum of particles of the five added polymers. We use the terms P, S2, and S3 to refer to mean aggregated results from samples P1–P3, S2.1–S2.4, and S3.1–S3.2, respectively. For results derived from Raman spectroscopy, only size classes ≥10 μm were considered to increase comparability to FTIR results, which were left untruncated.

In the absence of an unbiased reference value for the MP particle numbers in the samples, we used the arithmetic mean and standard deviation between participants as measures of centrality and agreement. To evaluate the deviation of the *i*^th^ participant’s result (*P*_*i*_) obtained for a sample from the overall mean of the results reported by all participants for that sample, we calculated a “relative deviation from the mean” (RMD, eq 1) where *P̅* denotes the overall sample mean.
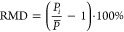
1

Using the relative standard deviation among participants (RSD, eq 2), we compare the dispersion of reported results between the different ILC design concepts.
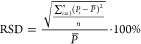
2

Due to the low number of participants using Raman (*N* ≤ 4) and FTIR (*N* ≤ 3) spectroscopy, mean differences between these groups were evaluated by one-sided, nonpaired, exact permutation tests^33^ and accepted as statistically significant for *p* ≤ 0.05, i.e., the proportion where the mean of values assigned to the lower group was greater than the mean of values assigned to the higher group in not more than 5% of all possible permutations.

Results were regarded as statistical outliers where experimental values were more than 1.5 times the interquartile range below the first or above the third quartile of all participants. They were excluded where technically justified, i.e., where a limited compatibility between the properties of a certain ILC sample and the applied measurement technique was identified.

The stability of a sample was evaluated by comparing the TMP measurements, conducted by the same participant, at the beginning *x*_pre_ and at the end *x*_post_ of the serial ILC. We used a stability criterion *c* (eq 4) in adaptation from ISO 13528.^15^ Here, we used the ratio between the “two-sided” relative pre/postdeviation Δ_rel_ (eq 3) and the RSD among the participants as a stability criterion, accounting for the fact that samples with multiple measurement areas may not have comparable absolute MP particle numbers. “Two-sided” here indicates that we take the double of the difference from the mean as the expected deviation range would be centered around the mean going into positive and negative directions.
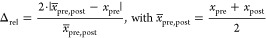
3
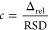
4

Acceptable stability was expected when *c* ≤ 0.3. When the calculated stability was only marginally above this threshold, *c* had to be corrected to include the measurement uncertainty (*u*) according to eq 6 as recommended for testing against the same threshold.^15^ We determined *u* as the average relative distance of *r* replicate postmeasurements from their mean (eq 5).
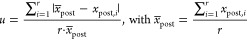
5
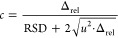
6

A spreadsheet file that was used for the calculations and figure generations and a Jupyter notebook containing the calculations of the permutation tests are provided in the data deposit.^34^

## Results and Discussion

**Figure 3:**
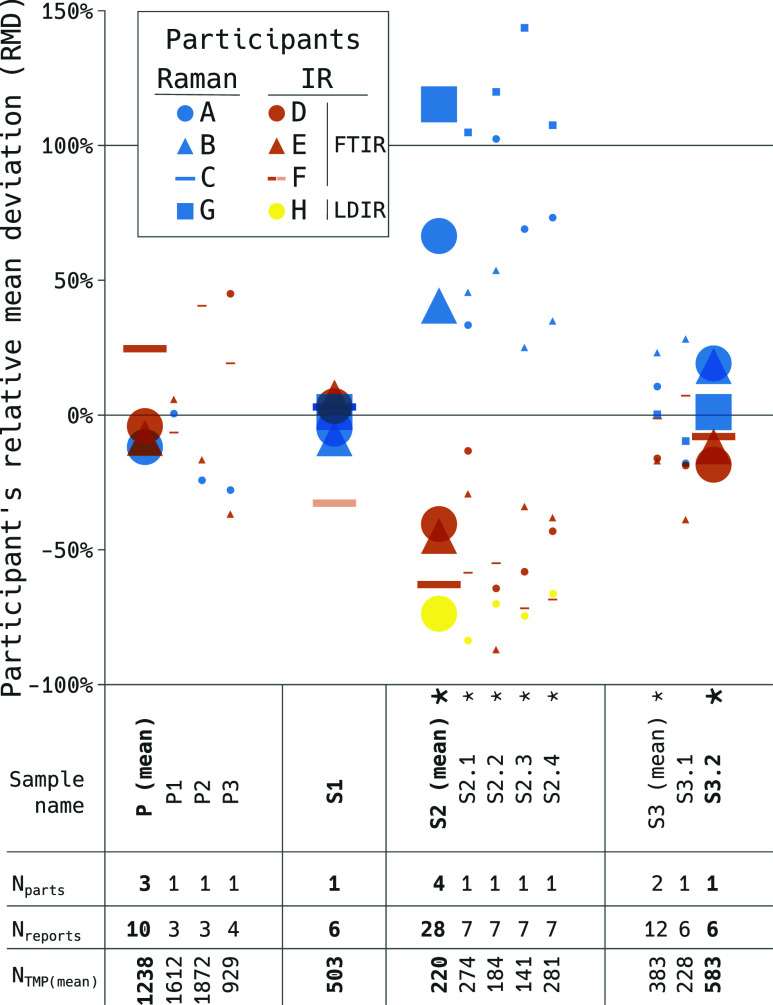
Particle numbers shown as percentage deviation from the participant mean per sample (RMD). Large symbols show the most important results per sample type. These are the pooled results for P and S2 where multiple sample parts were available and the single sample results S1 and S3.2. Sample S3.1 needed to be excluded for lack of stability. Small symbols indicate the RMDs for the constituting sample parts. Asterisks indicate significant differences between the subgroups’ mean RMDs (Raman vs IR, see also Table 2 for references of the techniques used by each participant). Participant F’s result for sample S1 was excluded as a statistically and technically justified outlier, and it is shown here semitransparently for reference only (not included in the mean calculation). Key data of the samples are provided below the horizontal axis. Sample name: terminology used according to Table 1 (P: samples used in parallel ILCs; S: samples used in serial ILCs). *N*_parts_: number of parts that were pooled under this sample name. *N*_reports_: number of reports returned by participants (for brevity showing the sum of reports in multipart samples). *N*_TMP_: reported particle numbers of TMP as mean over participants (polymer and participant-specific results are provided in the data deposit^34^). Absolute particle numbers are shown in Figure SI4.1.

We report on a novel immobilization technique for MPs and its application in serial ILCs compared to parallel ILCs. As absolute numbers of particles varied significantly between samples, we show the numbers of MP particles reported by the participants here relative to their mean (Figure 3), but raw count data are presented as bar charts in the Supporting Information (Figure SI4.1).

**Figure 4:**
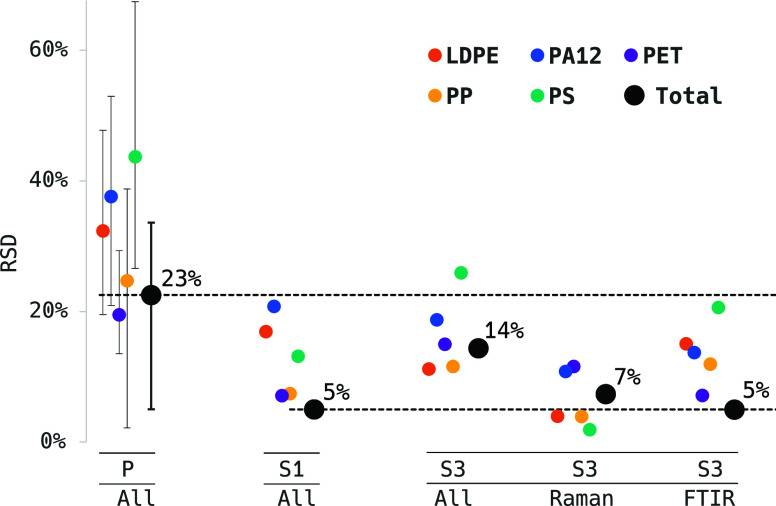
RSD among participants for the different ILC samples. Dashed lines indicate the RSD levels of TMP in the serial ILC of S1, which was 77% lower than in the parallel ILC design (results of samples P1, P2, and P3 were pooled as “P”). Whiskers show the extents to the minimum and maximum of the unpooled RSD values. RSD values of S2 can be seen in an extended version in the Supporting Information (Figure SI4.2). P, parallel samples; S, serial samples.

The serial ILC design, by isolating the specific measurement uncertainty, significantly reduces observed variation among participants compared to the parallel ILC, with a 77% lower RSD in the small-scale ILC comparison we conducted here (Figure 4).

### Isolation of the Specific Measurement Uncertainty by MP Immobilization

The parallel ILCs resulted in an RSD of 23% as derived from the mean of the three suspended samples, following Table 1 (pooled as sample P in Figure 4). The RSD spreads from 5% (P1) to 34% (P3) and is shown as whiskers in Figure 4. The high RSD, along with the absence of a consistent pattern among participants over the three samples (see Figure 4, P1–P3, for details), suggests that uncertainty sources like sample production, transport, and preparation for spectroscopy (see L0–L2 in Figure 1) may have contributed an important share of the overall variation of the results. Hence, the magnitude of the specific measurement uncertainty (L3–L5, Figure 1) cannot be estimated. The serial ILCs, with particles immobilized on the measurement substrates, exclude production- and preparation-derived uncertainties by design, as every participant measures the same sample. Thus, the specific measurement uncertainty can be investigated independently.

The S1 series on the 10 μm Si filter substrate resulted in an RSD of 5% (with participants A–E and G, Figure 4), giving an indication of the specific measurement uncertainty within the ILC consortium. It is observed that, generally, individual polymers have a larger RSD than TMP. The TMP individual values reported by the participants vary with RMDs between −9 and +6% around their mean (Figure 3). There was no significant difference between the RMD means of the ad hoc Raman and Fourier transform infrared (FTIR) subgroups for S1.

To test whether the serial ILC concept can be extended to other applications, a smaller pore size Si filter (S2) and aluminum oxide as another common filter substrate (S3) were explored in further ILCs. While the FTIR participants reported largely consistent results for the Al_2_O_3_-based sample S3, participant F reported difficulties in obtaining a reliable measurement result for the Si sample S1. Their analysis technique is based on a machine-learning polymer recognition model trained on Al_2_O_3_ substrates, which can be expected to have limited compatibility with the Si substrate. This underlines the importance of taking filter substrate preferences into account when designing an ILC. The use of different filter substrates can help to make an equitable assessment.

The overall RSD of S3 is 14% with regard to all participants (A, B, and D–G). However, when separated by the spectroscopic technique, the RSD is comparable to what has been described for S1: 7 and 5% for Raman and FTIR spectroscopy, respectively (Figure 4). Raman-based measurements of S3 have, on average, a significantly higher RMD of 25 percentage points (%pt) difference, significant at *p* ≤ 0.05 (Figure 3) compared to FTIR-based techniques. The other filter S3.2 is excluded from analysis as a substantial particle loss of 21% has been recorded between pre- and postmeasurements of the host participant, indicating that the immobilization was not sufficient (see also Figure 6 and Stability of Filtration-Immobilization).

For the 1 μm Si filter (S2), an even stronger segregation can be observed with respect to the spectroscopic technique. On average, Raman participants are >120%pt higher in terms of RMD compared to FTIR and LDIR. Accordingly, the RSD is substantially higher: 71% for TMP and up to 91% for the individual polymers, exceeding the RSD of the parallel ILC. In order not to distort the scale of Figure 4, S2 was omitted there. An extended version including S2 is presented in the Supporting Information (Figure SI4.2). We assume that a reason for the observed systematically lower TMP estimates of the FTIR and LDIR participants for the 1 μm filter S2, and, although weaker, for the Al_2_O_3_ filters S3 (see also Figure SI4.1), is likely an excess of potassium silicate adhesive, which remains in the smaller pores due to capillary forces and thus adversely affects the FTIR signal intensity. Further, the properties of Si wafer filters like material thickness and pore diameter, pattern, and distance could cause interferences in the IR wavelengths.^30^ In combination with the potassium silicate adhesive, this may have reduced polymer recognition for IR-based techniques. This was indeed the case when considering the LDIR transflectance measurements as the spectra of many particles appeared “blurred” or distorted due to the partial coverage by the adhesive. In transflectance, the IR light interacts twice with the surface of the particles plus with the reflecting substrate.

**Figure 5:**
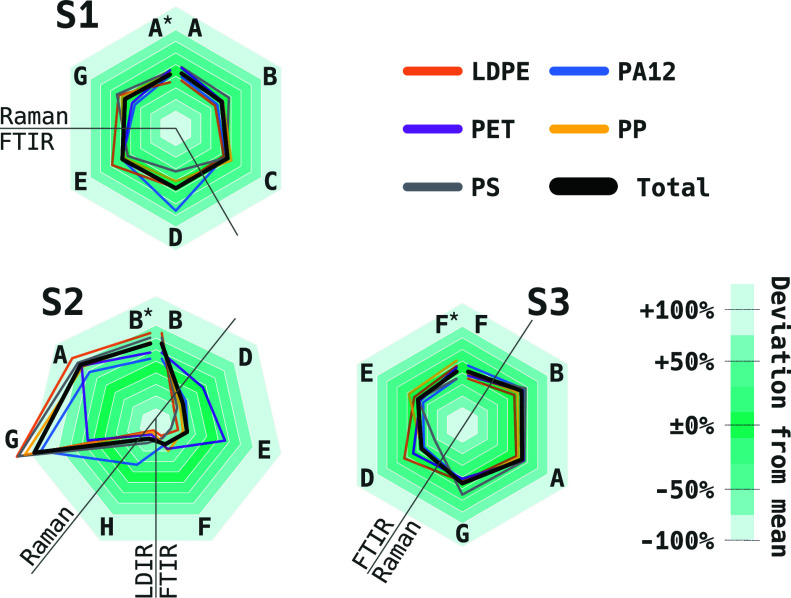
Reported results of the three serial ILC trials with samples S1–S3 as percentage deviation from the overall participant mean. Entries marked by asterisks indicate the post-transport control measurement of each ILC sample and do not constitute an own participant. Hairlines delineate the ad hoc subgroups of Raman, FTIR, and LDIR measurements.

Radar charts in Figure 5 show the RMDs of the participants for the five polymer types. The closer the lines are to the most intense color region (RMD ± 10%), the higher the consensus within the consortium. The host or initial participant is located at the top of the polygon, and the other participants follow clockwise in the order the samples were sent around. The trials finish with remeasurement by the host (marked with an asterisk in Figure 5) to determine the immobilization stability. The remeasurement results were not included in the mean for the RMD calculations.

S1 showed high consensus among the participants (Figure 5). Overall, we conclude that the use of the 10 μm Si filter, in contrast to 1 μm pore-sized filter S2, allowed better comparability between Raman and FTIR results. We attribute this observation to the resulting size range of particles (excluding the sub-10 μm fraction), which is better suited to the typical limits for FTIR, and to a lower capillary retention of the potassium silicate adhesive in the larger pores of S1.

As to the polymer type, the largest singular positive deviation from the overall participants mean in S1 occurs for the polyamide PA12, which has been identified as a software-caused false-positive problem by participant D. The machine learning-based analysis was sensitive to even weak presence of bands in the natural amide region, leading to pixels being erroneously classified as polyamide. This illustrates that the isolation of the specific measurement uncertainty achieved herein enables certain analysis pipelines to be debugged.

Neither parallel nor serial ILC designs can determine the absolute truth of the particle number present. This is clearly so when particle sizes get close to the instrumental detection limits, as it was the case here, at least for FTIR and LDIR at around 10 μm. Nevertheless, when only larger sizes are considered, agreement might be possible and very relevant. At least for the immobilized design, a theoretically viable—but very time-consuming—way to obtain an accepted true reference value would be an analysis with individual optical microscopy and manual spectroscopic assessment of all immobilized particles in a target area of a sample. However, even then, it would be difficult to achieve absolute certainty about the number of MP particles present. In borderline cases, it may be impossible even for human assessors to make a final decision about the extent of a single particle, because MPs do not always exhibit unequivocally visually separable particle boundaries.

The serial ILC performed here serves as a demonstrative proof of concept for the idea of using immobilized samples in serial ILCs. We recommend for benchmarking or standardizing ILCs, prepared in this manner, to use replicated samples as well as repetitions of the pre- and postmeasurements (*n* ≥ 3). They serve as a control of successful immobilization and allow to estimate the intra-analytical measurement uncertainty. Furthermore, the number of participants was low and the apparent particle numbers varied between the different ILC types (see also the table within Figure 3). Presenting more equal particle numbers to a larger group of participants will lead to increased stability of the uncertainty estimations that can be attributed to the applied analysis techniques.

### Stability of Filtration-Immobilization

The development of the immobilization method in the present study was adapted from a previous approach where spin-coating was used to distribute an adhesive on a carrier.^17^ The change to the filtration-immobilization principle proved advantageous as the amount of residual adhesive on the substrate surface is substantially reduced. We provide insights of spectroscopic effects of the remaining adhesive in SI5.

**Figure 6:**
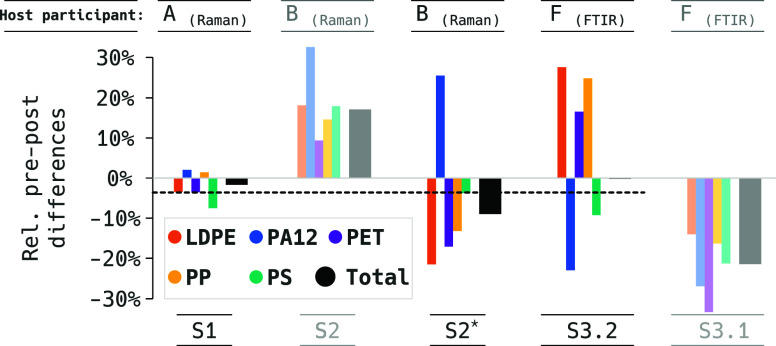
Relative differences of particle numbers per polymer identified by the host participant before and after the serial ILC. S2* was calculated only on the particle fraction ≥20 μm due to the reasons explained in the main text. The dashed line indicates the change in particle counts as the average “pre/postdifferences” of those samples with acceptable sample stability, i.e., only opaquely plotted bars. Note that the value for total MPs was very low in S3.2 (bar not visible).

In the serial ILC concept, the host participant repeats their measurements after the ring trial completion to ascertain whether particle loss occurred (Figure 6). Negligible differences indicate the adequate stability and permanence of the immobilization.

Participant B, who made the pre/postcomparison of sample S2 using Raman spectroscopy, received a software upgrade from the manufacturer between the two measurements that improved the instrument’s ability to detect smallest particles. The stability of the immobilization was therefore only evaluated on the ≥20 μm particle fraction of the pre- and postmeasurements. The comparison including particles between 10 and 20 μm is presented for reference (Figure 6) and shows a spurious increase in the abundance of particles in the postmeasurement.

In adaptation of the ISO 13528^15^ stability criteria *c* (eqs 4 and 6) to the serial ILC, we observe that samples S1, S2, and S3.2 qualify for being included in the ILC results. Their relative pre/postdifferences are within the threshold for acceptable stability of *c* ≤ 30%, namely, at 25, 14, and 1% of the interparticipant RSDs, respectively. For sample S1, this result was obtained using eq 6 with an expected measurement uncertainty of 0.056 and would have been 32% when using eq 4.

In addition to actual changes in particle numbers due to sample transport, deviations between pre/postmeasurements are also influenced by the expected—and here unknown—intra-analytical variance of the host participants. However, the overall low pre/postdifference (Figure 6; dashed line: −4% on average of the samples accepted according to the “criterion *c*”) implies that only relatively small intra-analytical variances are to be expected here.

There was an apparent difference of immobilization stability between the two Al_2_O_3_ filters (S3.1 and S3.2). In contrast to the Si filters, a rinsing procedure to remove insufficiently immobilized particles after curing was not applied due to the fragility of the material. Future studies aiming for immobilized samples on alumina membranes should explore other solutions for removing loose particles.

Pre/postdifferences in the individual polymer types appear to largely cancel out each other in TMP, which is especially obvious for samples S2 and S3.2. This indicates a differential polymer type misclassification problem, not only between participants (Figure 4) but also intra-analytically. This reveals an interesting research question on whether there is in general a significantly lower uncertainty on TMP counts than on quantifications of individual polymers.

### Perspective of Environmental Particle Evaluation

Below, we summarize the lessons learned from the interlaboratory trials presented here and give recommendations to encourage much needed further methodological advancement in this area. The approach we present here is targeting the size range of about 10–100 μm. It would likely be suitable for MPs up to about 500 μm, but the effectiveness of the filtration immobilization is yet to be evaluated in this size range. As such, we only cover a part of the environmentally relevant MPs. For larger sizes, simpler methods like obtaining a true reference value by manual counting become viable and are therefore likely to be preferred.

With sample S1, we found a 10 μm filter well suited for comparative measurements with Raman, FTIR, and LDIR spectroscopy, while the 1 μm filter S2 posed several obstacles to FTIR and LDIR detection. This includes a higher presence of particles in size ranges where physical limitations restrict successful detection by these techniques. In Raman spectroscopy, potassium silicate shows distinct bands at 545 and 1029 cm^–1^ (Figure SI5.1), but they did not interfere with the polymer classification, primarily because Raman spectroscopy is acting on the up-facing surfaces of the particles, where little or no excess adhesive is expected. For FTIR and LDIR spectroscopy measured in transmission and transflectance modes, respectively, any adhesive between the particles and the filter surface or inside the filter pores will interact with the IR light. However, this will not result in conjugate spectra (i.e., polymer spectra clearly superimposed inorganic bands of the adhesive) because the FTIR measurements for polymer identification were made against a background of potassium silicate on the filter material. Instead, the IR adsorption of the silica compounds manifests itself as increased noise because it limits the IR energy that can reach the detector. Regarding the concentration of potassium silicate, we chose the lowest tested concentration as the best option. It seems reasonable thinking that further dilutions may be a promising endeavor to decrease potential impacts to certain techniques.

Many analytical protocols employ subsampling measurement approaches due to instrumental or computational limitations or to reduce measurement time. This entails extensive extrapolation of the actual measurement result to the whole sample, contributing to the uncertainty propagation. In fact, subsampling approaches (see also Figure 1) have been demonstrated to cause substantial errors in the determination of the total particle numbers.^25^ A major advantage of the immobilized serial ILC design is that such a subsampling error is eliminated by the precise definition of a commonly measured area. Here, the laser engraving technique developed for Si filter S2 proved to be highly useful in that it expedites and unifies measurements and result evaluations. Due to the particles being removed from the interstice, minor unavoidable differences in the size and positioning of the measurement areas will not significantly affect the results when all participants select areas slightly larger than the visible target areas.

For a permanently immobilized serial ILC sample, we consider three properties to be most desirable:

- The substrate and immobilization achieve sufficient stability, i.e., they withstand the repetitive handling, transportation, and measurement.
- Its physical properties do not interfere with or discriminate against certain measurement techniques, e.g., sufficient IR transparency is warranted.
- The sample allows for some form of return to original state, e.g., by rinsing off any adherent contaminating particles prior to each participant’s measurement.

Compared to the creation of numerous sample specimens for parallel ILCs, the immobilized approach requires less strict contamination control during production. Contamination during sample generation is excluded by design as everything that gets immobilized becomes part of the sample. It is still advisable to prepare samples under reasonable analytical laboratory conditions to avoid unintentionally introduced particles, producing spurious results.

### Outlook and Future Perspectives

The immobilized filters in our serial ILC were prepared at small research laboratory scale. While offering significant advantages over traditional parallel designs, they are not intended as a permanent solution. They serve as an intermediate step in establishing robust analytical protocols and identifying sources of measurement uncertainty. However, the concept should be the taken up for the development and supply of certified reference materials (CRMs) that can be routinely used for method validation and quality control in laboratories worldwide. CRMs, produced and certified by accredited organizations such as the European Joint Research Centre (JRC), the German Federal Institute for Materials Research and Testing (BAM), the National Metrology Institute of Japan (NMIJ), the International Atomic Energy Agency (IAEA), or the United States National Institute of Standards and Technology (NIST), provide a standardized and traceable means of validating analytical methods and ensuring interlaboratory comparability. For MPs, however, the provision of CRMs presents unique challenges due to the particulate nature of the analyte. Collaboration between research institutions, regulatory bodies, and accredited reference material producers is essential to overcome these challenges and establish commercially available immobilized and suspended CRMs for MP analysis.

With immobilized MP CRMs available, automated reproducible measurements may be set up with minimal manual involvement. These procedures can run routinely on a periodic schedule or after any changes in measurement protocols, equipment, or personnel. Over time, the results can become highly valuable for the laboratory to monitor the relative analytical proficiency and spot any arising problems in MP detection.

Further questions should be addressed to establish a broader applicability of the immobilized samples. For instance, knowledge on immobilization stability of larger MP sizes (e.g., 100–1000 μm), fibers or natural particulates, may allow for an application also to field samples. This can reduce particle loss or disarrangement in the transition from sample preparation to spectroscopic measurement. Additionally, it may be used to build up persistent sample archives for repeatable measuring. At the current stage, however, we do not recommend the immobilization technique for quantitative sample preservation. We included here steps of sample flushing to remove incompletely immobilized particles, which would obviously not be an option in field samples intended for MP quantification. Provided that the objective is to conduct an ILC or similar experiment—where the total number of MPs present in the sample prior to immobilization is not of interest—we may reasonably assume that the technique would work just as effectively if MPs from the environment were used instead of the milled and sieved pristine particles employed in this study.

Our results suggest that other research on particulate pollutants beyond like fly ashes, cement dust, soot, or spores can benefit from adapting the immobilization technique to yield reproducibly measurable samples. In particular, further developments targeting immobilization of atmospheric particulate matter samples will help in comparability studies of microspectroscopic and mass spectroscopic analyses^35,36^ (see also SI6). Likewise, other multianalysis studies, such as correlative microscopy or spectroscopy, could benefit from enhanced precision and accuracy when working with particle immobilizations.

## Data Availability

Data of measured MP particles per sample and participant with statistical analysis and raw version of the figures created thereof are provided as a Microsoft Office Excel spreadsheet in the data and code deposit^34^ at 10.5281/zenodo.10791088.

## Supporting Information Available

Material and methodological details, additional microscopic and spectroscopic information, and an extended version of Figure 4 (PDF)
