# Supplementary material for: What Goes Around Should Not Move Around: Immobilizing Microplastics as a New Approach for Analytical Ring Trials
Source: Environ Sci Technol. 2024 Dec 3;58(50):22224–34. doi: 10.1021/acs.est.4c09427 (PMC11656703; doi:10.1021/acs.est.4c09427)
Supplement: Supplementary file 1 — es4c09427_si_001.pdf [file es4c09427_si_001.pdf]

Supporting Information

for the Paper

“What Goes Around Should Not Move Around: Immobilizing Microplastics as a New Approach for Analytical Ring Trials”

Robin Lenz[1,2,✉], Kristina Enders[1,2], Eva Cseperke Vizsolyi[3], Mareike Schumacher[1], Julia Lötsch[1], Martin Löder[3], Gabriele Eder[4], Yuliya Voronko[4], José Manuel Andrade-Garda[5], Soledad Muniategui-Lorenzo[5], Christian Laforsch[3], Dieter Fischer[1,✉] and Matthias Labrenz[2,✉]

[1] Leibniz Institute of Polymer Research Dresden, 01069 Dresden, Germany
[2] Leibniz Institute for Baltic Sea Research Warnemünde, 18119 Rostock, Germany
[3] University of Bayreuth, 95440 Bayreuth, Germany
[4] Österreichisches Forschungsinstitut für Chemie und Technik, 1030 Wien, Austria
[5] University of A Coruña, 15071 A Coruña, Spain
✉ Correspondence: Robin Lenz <robin.lenz@ipfdd.de>, Dieter Fischer <fisch@ipfdd.de>, Matthias Labrenz <matthias.labrenz@io-warnemuende.de>

Contents

SI1: 3
Preparation of particle suspensions P1 – P3 for the parallel ILC 3
Preparation of media for the immobilised MP samples S1 – S3 for serial ILCs 4
SI2: Approaches for measurement target area definition 5
SI3: Custom filter holder and transportation box 6
SI4.1: Absolute particle counts 7
SI4.2: Extended plot: relative standard deviations 8
SI5: Potassium silicate – spectroscopic effects 8
SI6: Perspective of mass-spectroscopic techniques 11
References 12

Summary:
Number of pages: 12
Number of figures: 7
Number of tables: 0

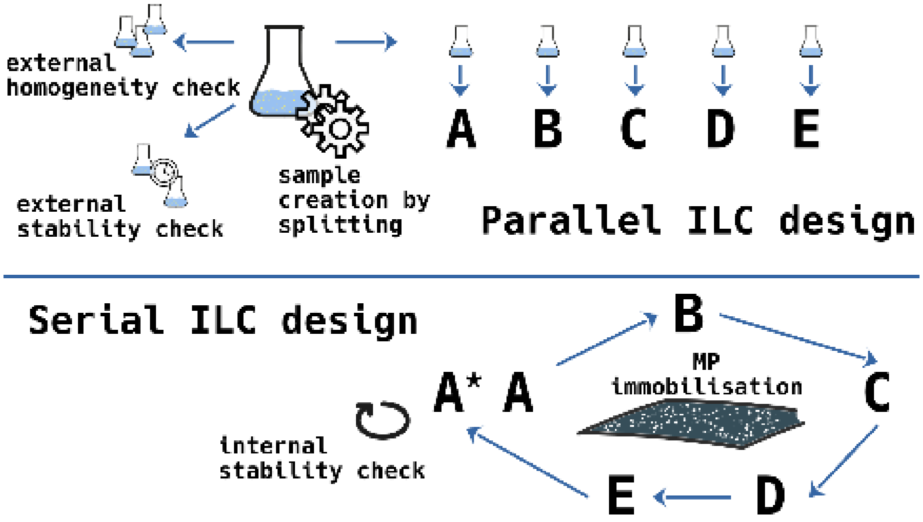

Table-of-Contents Graphic | A parallel and a serial ILC design with participating laboratories A - E. In a parallel ILC (upper part), a separate sample specimen is prepared for each participant, with additional ones held back for homogeneity and stability controls. Specifically for microparticulate analytes like microplastics (MP), the homogeneity of the measurand between sample specimen cannot be guaranteed or controlled. In a serial ILC (lower part), only one sample exists which is measured in turns by all participants, with host participant A repeating their measurement (here A*) to evaluate sample stability. This requires the preparation of a permanently immobilised sample. For its production we propose a filtration-immobilisation technique using potassium silicate as an inorganic adhesive. After particles are applied to the filter substrate as aqueous suspension, the curing of the adhesive arrests them in position by water-insoluble bonding to the substrate. Transported in suitable containers, these samples can be passed around the consortium of participants without compromising their integrity, allowing them to be measured repeatedly using different techniques chosen by the participants.

SI1:

Preparation of particle suspensions P1 – P3 for the parallel ILC

An aqueous solution of 0.005% (v/v) TritonX-100 (Merck KGaA) surfactant was prepared and filtered through 0.2 μm cellulose acetate filters to avoid microplastics (MP) contamination. 1 mg of each polymer (measured by an XPR6U microscale, Mettler Toledo, Switzerland) was added to 500 mL of the surfactant solution and homogenised in an ultrasonic bath. The particle suspension was filtered onto a 2 μm stainless steel filter (Spörl KG, Germany) and the bottle was rinsed out to avoid the loss of particles sticking to the inner walls. The filtrate, containing the particle fraction below 2 μm, was discarded. During filtration, care was taken to ensure a homogenous distribution of the particles on the filter. The sample was then halved using an in-house made sample dividing device, which is able to support the filter from below while covering one half of the filter (see Figure SI1). With this tool, it is possible to wash off the uncovered half of the filter into a glass beaker with 500 mL of the MP-free surfactant solution. The technique was developed to provide a more homogenous splitting compared to pipetting the suspension, where MP can attach to the pipette tips inside and outside, and where inhomogeneities of the polymer distributions may arise due to different material densities, sizes and shapes. We note that the use of this specific tool may still have had an influence on the resulting MP content of the splits obtained. However, the same would be true if any other splitting technique was chosen. In parallel ILCs, the uncertainties of sample production will always propagate to the final results (as we illustrate in Figure 1).

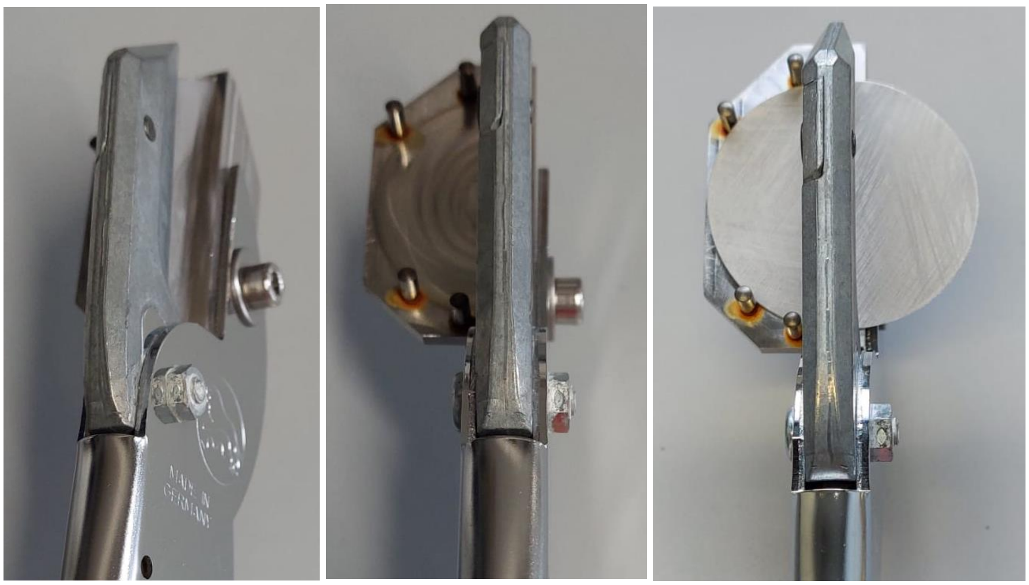

Figure SI1 | Modified pliers used as a sample splitting device. The right image shows how a 47 mm diameter stainless steel mesh filter is inserted. The device supports one half of the filter, blocking one half by a separating wall while particles on the other half of the filter can be rinsed off freely.

The procedure of filtration and splitting was conducted five times consecutively, in order to yield suspensions, where the particle concentration was in an acceptable range. The suspensions were then transferred into glass Erlenmeyer flasks and closed with glass stoppers using metal clamps to secure the suspensions during transport. The participants were advised to filter the sample specimens onto a spectroscopic substrate of their choice: either Si porous wafer filters or alumina membranes were used.

Preparation of media for the immobilised MP samples S1 – S3 for serial ILCs

For immobilisation, we used a technical grade potassium silicate solution ($K_2SiO_3 \cdot n\ H_2O$, also known as "water glass", 1.25 g cm^{-1}, Baufan Bauchemie Leipzig GmbH) as inorganic adhesive bonding agent, which is non-toxic, readily available and inexpensive. When exposed to air, the water evaporates from the aqueous potassium silicate solution, which then silicifies by absorbing CO_2. We use this effect to form polymeric silica structures on the contact surfaces between the MP particles and the filter substrate. This eliminates the need for organic polymer-based adhesives, which have been used to immobilise MP particles but introduce interfering bands[1]. Therefore, we avoid that polymer-based adhesive bands overlay the MP spectra obtained for particle identification. Based on preliminary tests with potassium silicate concentrations of 10%, 20% and 30% (v/v), it was found that 10% gave the best trade-off between filterability, immobilisation capabilities, layer thickness and pot life. Higher concentrations led to increased amounts of residual adhesive on the immobilised particles, which disturbs in particular FTIR transmission measurements. An attempt to create K_2SiO_3-based immobilisation by spin-coating[1] did not produce satisfactory results here because of uneven wafer surface wetting and crack formation during curing.

For samples S1 and S2, an adhesive stock solution was prepared by mixing 10 mL of a potassium silicate solution with 90 mL MP-free H_2O at room temperature in a glass Erlenmeyer flask with glass stopper. The solution was then filtered through 1 µm Si filters. The filtrate was used as the MP-free suspension and adhesion medium thereafter. A total mass of 1.52 mg of a powder mix of approximately equal bulk volumes of the five polymers (LDPE, PA12, PET, PP, PS) was added to the prepared medium. The closed flask with the suspension was submitted to an ultrasonic bath (10 min, USC600TH, VWR, 45 kHz, 120 W) for particle dispersion and then held on top of a high frequency excentrical shaker for homogenisation while volumes were being extracted for filtration using 1 mL Pasteur glass pipettes.

For samples S3, the same adhesion medium was used, however, without added MP particles.

SI2: Approaches for measurement target area definition

1. For sample S1, participants were given a step-by-step guideline to find the specific area based on a macroscopically clearly visible feature on the filter surface (Figure 2a in the main text). The rectangular area dimensions were 2.2 by 2.0 mm.

2. For sample S2, four target areas were created using an engraving laser. Through the engraving process, we produced a grid of four independent measurement areas (A1 - A4, 2 by 2 mm each) separated by a frame where the laser had burnt off the top layer including the MP particles, the adhesive and approximately 3 µm of the Si filter material. Scanning electron microscopy (SEM) images were obtained on a Zeiss Ultra Plus SEM at 3 kV after sputtering 3 nm platinum on a replicate of S2 which was not used for the spectroscopic measurements (Figure 2b in the main text).

   The engraving was conducted using the following equipment and settings:

   Instrument: Rofin Combiline Advanced WT
   Laser source: Rofin Powerline F20 Varia
   Wave length: 1064 nm

   Process parameters for used in the engraving of sample S2:

   Track width: 0,015mm
   Laser power: 0.8W
   Frequency: 20000Hz
   Speed: 50mm/s
   Pulse width: 4ns
   Defocussing: 0%

   Exemplary CAD files are provided as part of the data deposit[2].

3. On the Al_2O_3 filters (samples S3), before particle immobilisation, a boundary was drawn manually with a thin black marker pen, the inside of which was the intended measurement target area (shown in Figure 2c in the main text). Although irregular, these manually defined measurement areas were approximately rectangular with dimensions of 1.9 by 1.8 mm and 1.8 by 1.6 mm for S3.1 and S3.2, respectively.

SI3: Custom filter holder and transportation box

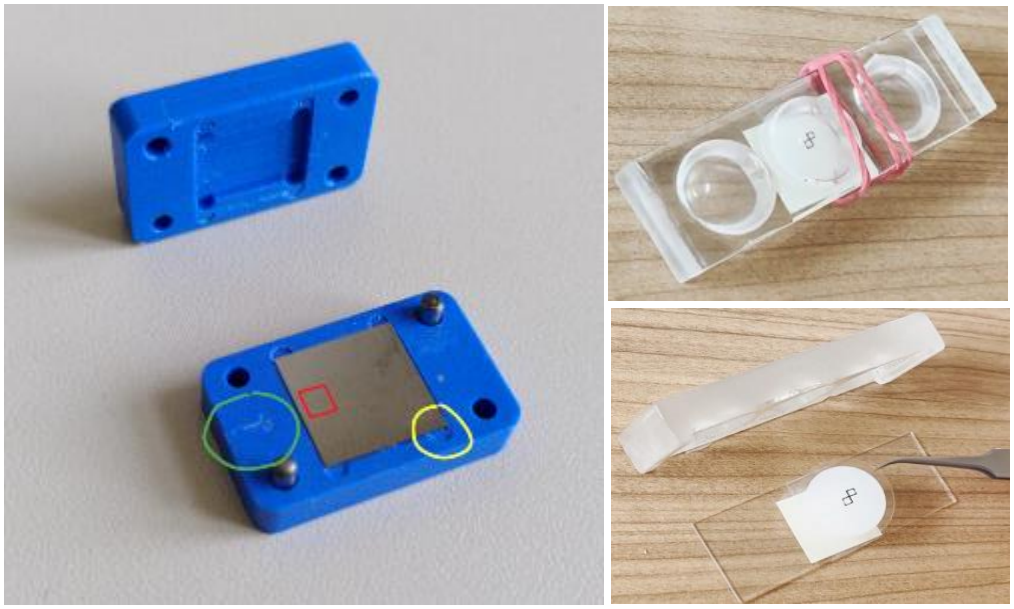

Figure SI3 | Custom-made Si filter holders for sample transport. Left: blue PTFE containers used for sample S1 and S2 consist of two parts that can be plugged together. During shipping the holder was tightly wrapped in adhesive tape to ensure that the sample remained in place. The engraved arrow on one side of the bottom part (green circle) indicates where the measurement area should be located (red square). This measure keeps the measurement area out of the filter support area where friction can affect the sample. There are also milled pockets in the corners for gentle handling of the filter with tweezers (yellow circle). Right upper: Sample S3 was transported in a glass sandwich with a microscopy slide as the bottom and a thicker circular concave cavity glass cover. When closed the slide slips in place into the recess of the cover and was secured with rubber bands. Right lower: After opening, the alumina filter was moved by tweezers on the outer support ring. The hand-drawn measurement area markings are visible.

The consortium consisted of participants from three countries (Austria, Germany, Spain). Immobilised samples of the two different substrates (Si and alumina) investigated here for use in serial ILCs were prepared by two different groups at different institutes. This includes the development of the transport solutions, which therefore also differ significantly. While the Si filters were shipped in a blue PTFE box custom-made by a scientific mechanical workshop, the alumina filters were secured for transport using only off-the-shelf laboratory glassware (Figure SI3).

SI4.1: Absolute particle counts

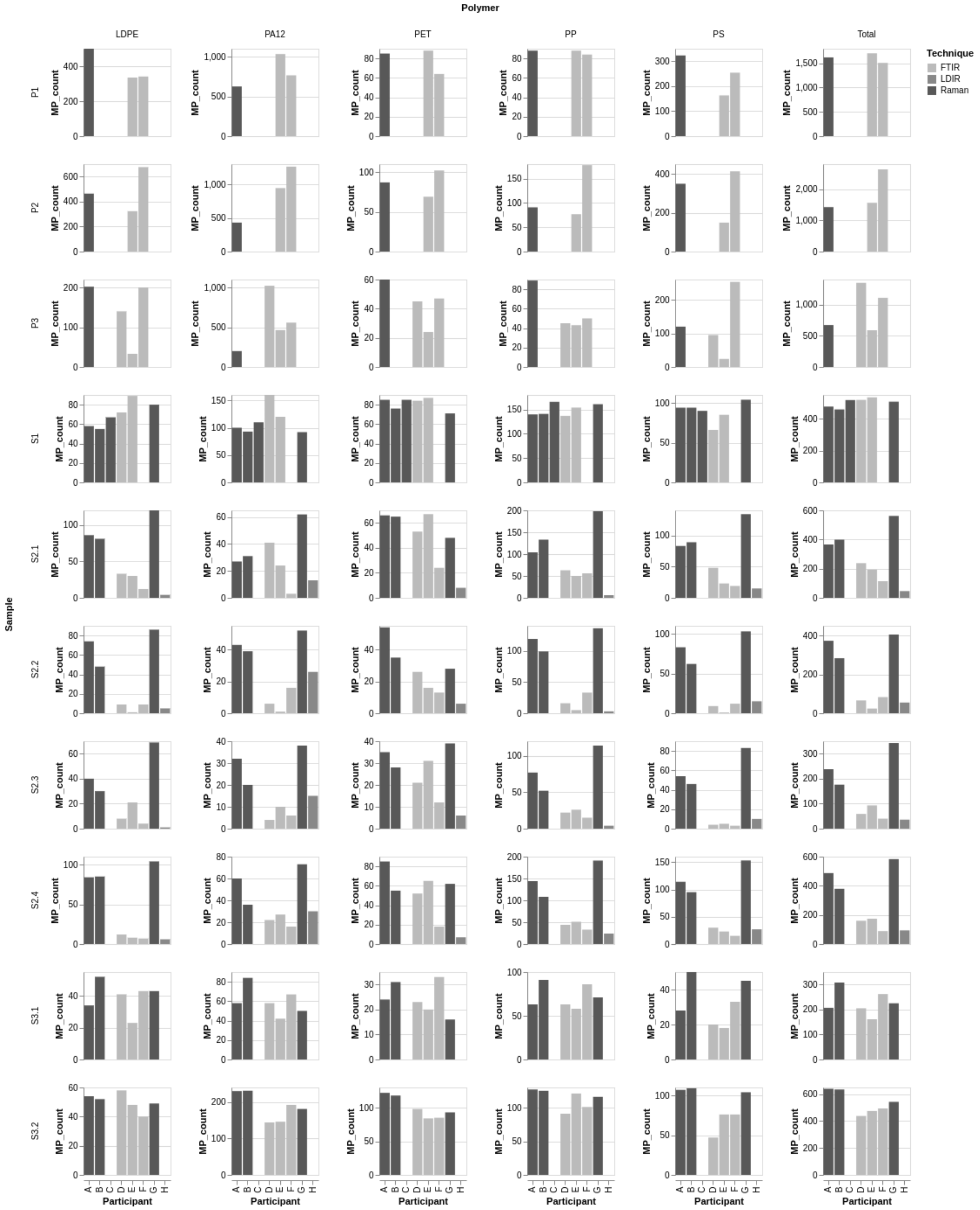

Figure SI4.1 | Counts reported by the participants, after harmonisation of common measured area and particle sizes (i.e. Raman ≥ 10 µm). Bars are missing where samples were not measured or results not reported.

SI4.2: Extended plot: relative standard deviations

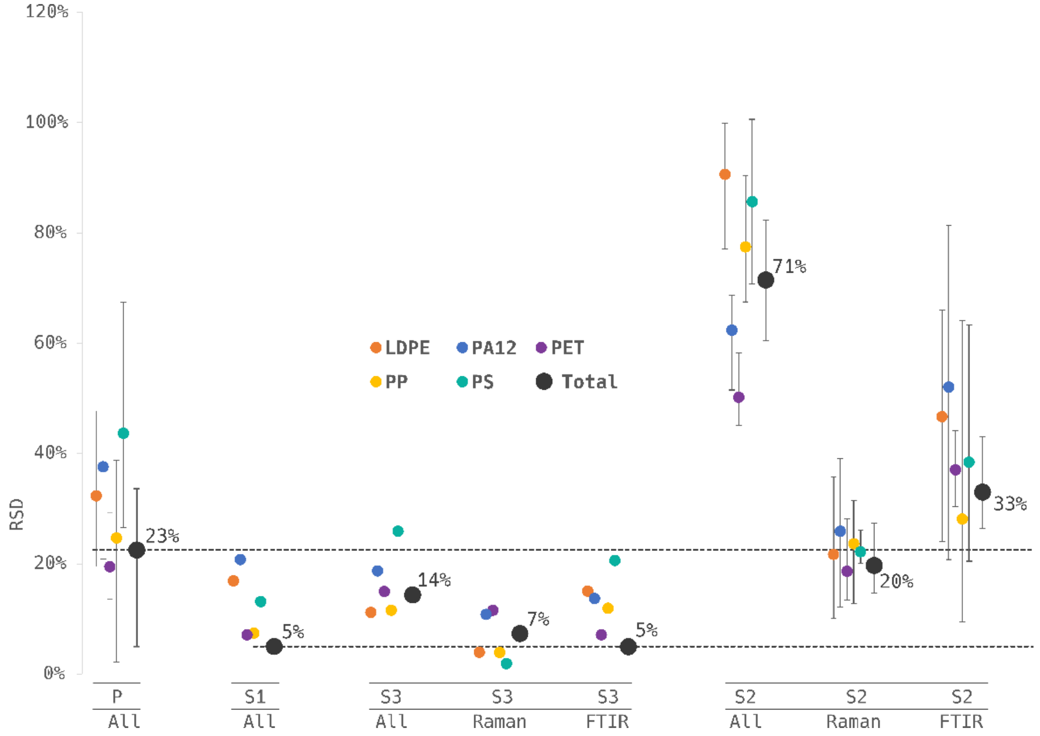

Figure SI4.2 | RSD among participants for the different ILC samples including sample S2. The greater RSD in S2 is explained by the surplus of potassium silicate adhesive remaining in the smaller pores and the resulting interference, which negatively affect IR-based techniques. LDIR (participant H) is only represented in the "All" category of S2. Whiskers show the extents to the minimum and maximum of the unpooled RSD values.

SI5: Potassium silicate – spectroscopic effects

Excess amounts of potassium silicate negatively impact the signal to noise ratio of FTIR and LDIR measurements (as has been observed on the smaller-pore sized filters S2 and partly S3). In Raman spectra it can exhibit bands around 545 and 1030 cm-1 (Figure S5.1), however, we observed no negative impact in the spectra acquired on the surfaces of the measured polymer types. For reference, potassium silicate spectra were recorded and are provided with the deposited data[2].

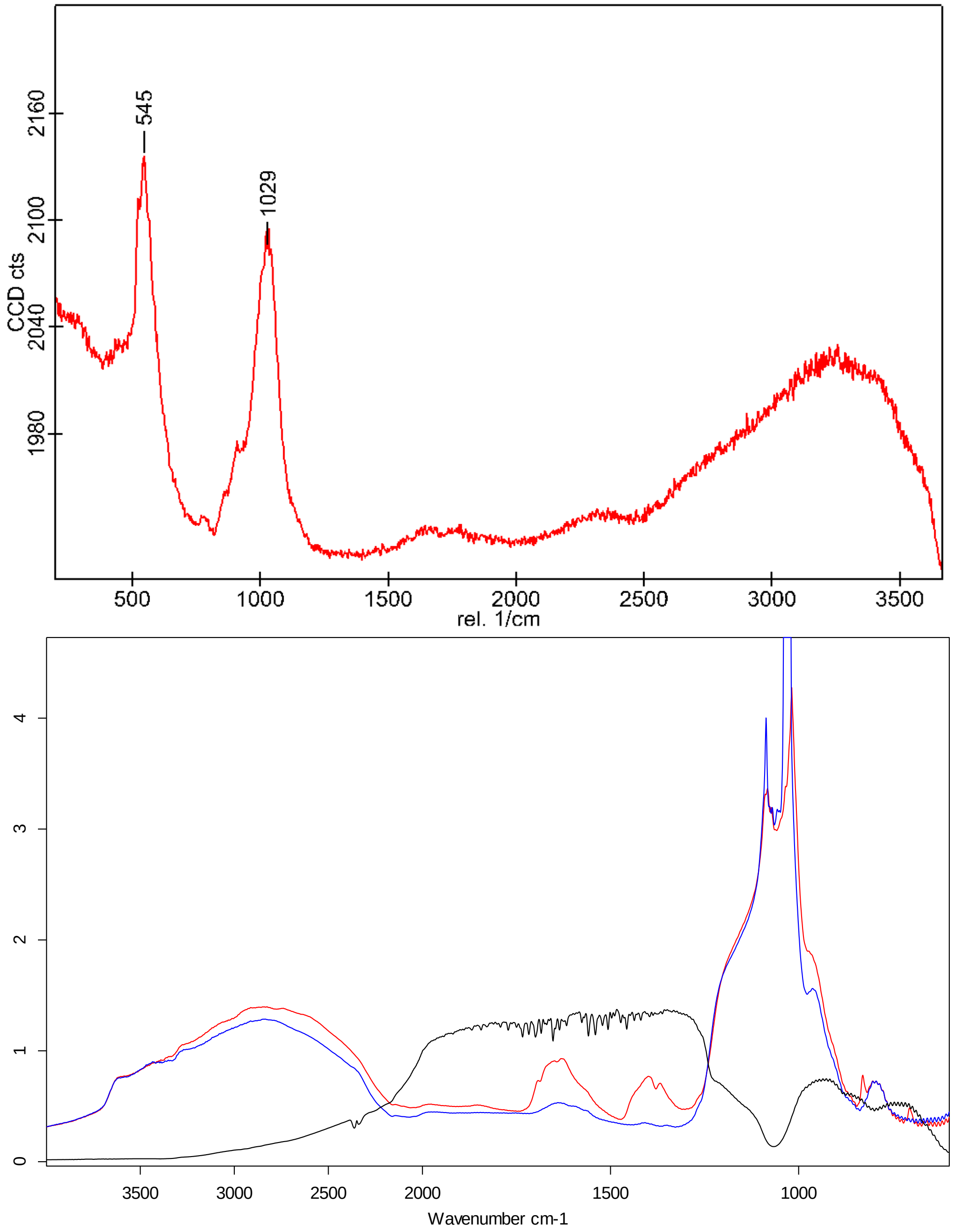

Figure SI5.1 | Raman and FTIR spectra of the polymerised potassium silicate, used as an inorganic adhesive for particle immobilisations. Water glass solution (Baufan Bauchemie Leipzig GmbH) was dripped onto an ethanol-cleaned Si wafer and allowed to dry. Upper: Raman spectrum, measurement conditions: 532 nm, 20 x, 50 accumulation, 0.5 s, 5 mW. Lower: FTIR spectra: in black shows the single-channel transmission spectrum of the Si filter without potassium silicate (used as background for the other two spectra), red and blue show absorption spectra of the silica-laden filter at locations with and without excess crusts, respectively. FTIR microscope Bruker Hyperion 2000, coupled to Vertex 70, 4000 – 600 cm^{-1}, HgCdTe detector, resolution 4 cm^{-1}, 100 scans per acquisition.

A deliberately exploited effect of the filtration immobilisation on the 10 µm pore size Si filter is the optimal distribution of particles on the substrate. Smaller particles predominantly come to rest on top of single pore cavities. This provides a nearly regular particle pattern which ensures that high particle

numbers can be measured in a small area. Larger particles are irregularly interspaced between or on top the grid-like smaller particles, leading to realistic scenarios of tightly aligned or overlaid particles. An example of a region of sample S1 illustrates this distribution (Figure S5.2).

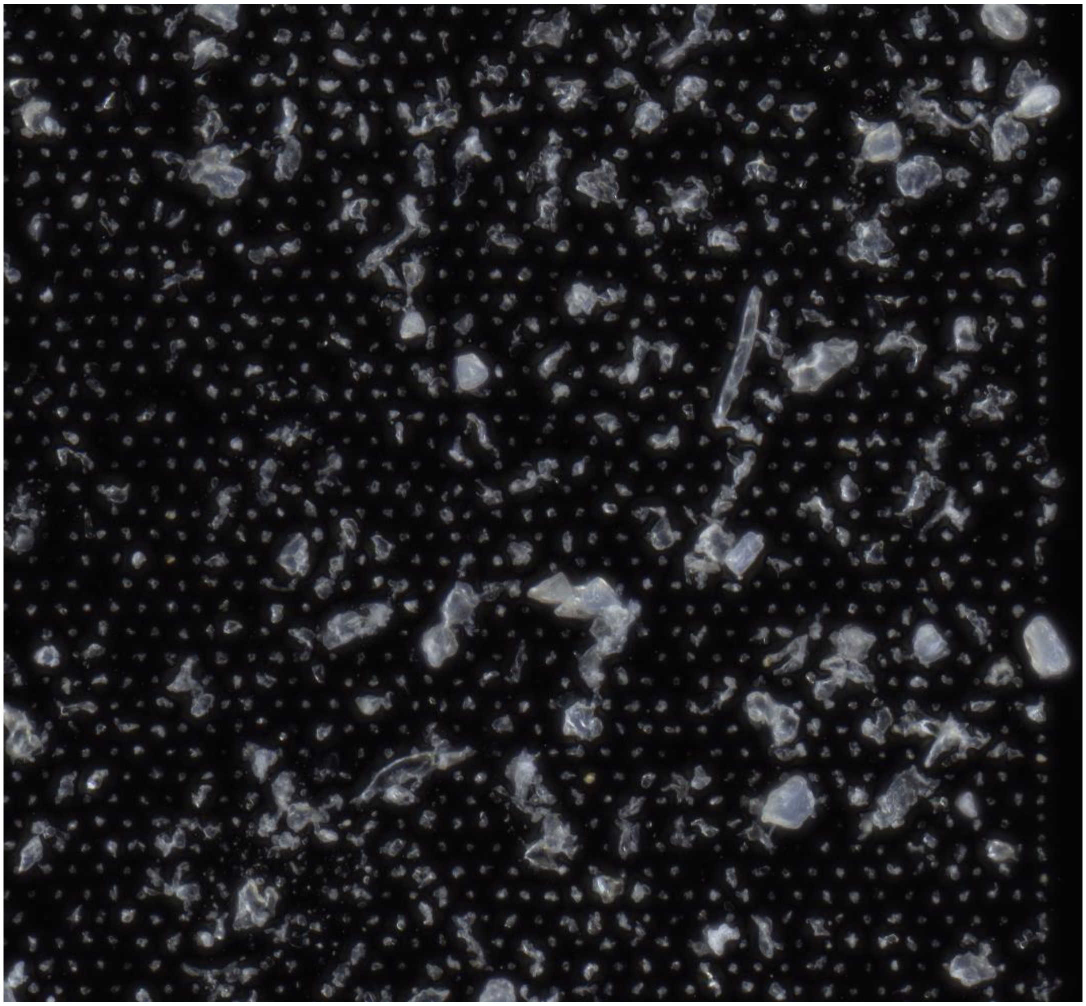

Figure SI5.2 | Measurement area of sample S1. The arrangement of particles is a result of the immobilisation filtration principle. Particles close to the pore size (10 µm) assemble in a grid like pattern on top of the pore cavities.

SI6: Perspective of mass-spectroscopic techniques

While we did not employ Pyrolysis-Gas Chromatography/Mass Spectrometry (Py-GC-MS) in this study, it is worth discussing its value, as it emerged as a valuable complementary technique for the analysis of MP[3–5]. Given an adequate pre-concentration of MP, so that the total mass of each polymer in a sample exceeds the respective limits of quantification, this technique is particularly useful for measuring small MP and nanoplastics embedded in complex matrices[3], such as soils and sediments, where traditional spectroscopic methods may face limitations.

Incorporating Py-GC-MS data would enable valuable comparative measurements between particle- and mass-based techniques. For instance, Raman spectroscopy, as employed by the participants in our ILCs, not only identifies polymer types but also provides high-resolution microscopic images that allow for particle size estimation in three dimensions. This capability enables the conversion of the obtained MP particle number to an estimated mass, which could be compared to Py-GC-MS results. Similar comparisons between FTIR and Py-GC-MS have been conducted[6,7].

However, Py-GC-MS is a destructive method which conflicts with the concept of a serial ILC that relies on a single sample being passed around among participants. One potential solution could be to include one Py-GC-MS participant as the last in the sequence of the ILC. This approach would allow for the comparison of mass-based and particle-based techniques, but it comes with the drawback that the host participant would not be able to repeat their measurement after the ILC for stability control. Additionally, the sample could not be preserved for possible future re-consultations. Therefore, in scenarios of mixed ILCs, including participants using mass- as well as particle-based techniques, a parallel design might still be preferable.

References

1. Lenz, R. *et al.* Measuring impacts of microplastic treatments via image recognition on immobilised particles below 100 µm. *Microplastics Nanoplastics* **1**, 12 (2021).
2. Lenz, R. *et al.* Data and supplementary files of the publication: "What Goes Around Should Not Move Around: Immobilizing Microplastics as a New Approach for Analytical Ring Trials". https://doi.org/10.5281/zenodo.10791088 (2024).
3. Fischer, M. & Scholz-Böttcher, B. M. Microplastics analysis in environmental samples – recent pyrolysis-gas chromatography-mass spectrometry method improvements to increase the reliability of mass-related data. *Anal. Methods* **11**, 2489–2497 (2019).
4. Ivleva, N. P. Chemical Analysis of Microplastics and Nanoplastics: Challenges, Advanced Methods, and Perspectives. *Chem. Rev.* **121**, 11886–11936 (2021).
5. Scholz-Böttcher, B. M. Mass-based methods for the analysis of micro- and nanoplastics. *Wiley Anal. Sci. Mag.* (2023) doi:10.1002/was.00050419.
6. Primpke, S., Fischer, M., Lorenz, C., Gerdts, G. & Scholz-Böttcher, B. M. Comparison of pyrolysis gas chromatography/mass spectrometry and hyperspectral FTIR imaging spectroscopy for the analysis of microplastics. *Anal. Bioanal. Chem.* 1–16 (2020) doi:10.1007/s00216-020-02979-w.
7. Primpke, S., Fischer, M., Lorenz, C., Gerdts, G. & Scholz-Boettcher, B. Quantifying Microplastics in Complex Environmental Samples: Py-Gc/Ms vs. Hyperspectral Ftir – Potential and Limitations. in *30th International Meeting on Organic Geochemistry (IMOG 2021)* 1–2 (European Association of Geoscientists & Engineers, Online, 2021). doi:10.3997/2214-4609.202134253.
